# Analyses of single marker and pairwise effects of candidate loci for rheumatoid arthritis using logistic regression and random forests

**DOI:** 10.1186/1753-6561-1-s1-s54

**Published:** 2007-12-18

**Authors:** Beate Glaser, Ivan Nikolov, Daniel Chubb, Marian L Hamshere, Ricardo Segurado, Valentina Moskvina, Peter Holmans

**Affiliations:** 1 Biostatistics and Bioinformatics Unit, and Department of Psychological Medicine, Cardiff University, School of Medicine, Heath Park, Cardiff, Wales, CF14 4XN, UK

## Abstract

Using parametric and nonparametric techniques, our study investigated the presence of single locus and pairwise effects between 20 markers of the Genetic Analysis Workshop 15 (GAW15) North American Rheumatoid Arthritis Consortium (NARAC) candidate gene data set (Problem 2), analyzing 463 independent patients and 855 controls. Specifically, our work examined the correspondence between logistic regression (LR) analysis of single-locus and pairwise interaction effects, and random forest (RF) single and joint importance measures. For this comparison, we selected small but stable RFs (500 trees), which showed strong correlations (*r*~0.98) between their importance measures and those by RFs grown on 5000 trees. Both RF importance measures captured most of the LR single-locus and pairwise interaction effects, while joint importance measures also corresponded to full LR models containing main and interaction effects. We furthermore showed that RF measures were particularly sensitive to data imputation. The most consistent pairwise effect on rheumatoid arthritis was found between two markers within *MAP3K7IP2/SUMO4 *on 6q25.1, although LR and RFs assigned different significance levels.

Within a hypothetical two-stage design, pairwise LR analysis of all markers with significant RF single importance would have reduced the number of possible combinations in our small data set by 61%, whereas joint importance measures would have been less efficient for marker pair reduction. This suggests that RF single importance measures, which are able to detect a wide range of interaction effects and are computationally very efficient, might be exploited as pre-screening tool for larger association studies. Follow-up analysis, such as by LR, is required since RFs do not indicate high-risk genotype combinations.

## Background

The analysis of genetic association studies for complex diseases requires the identification of significant single marker and interaction signals among a vast background of noise. In the presence of epistatically interacting loci, exhaustive searches of all possible two-marker combinations using the classic logistic regression (LR) approach have been shown to be more powerful than single-marker analysis in simulations [1], even when correcting for multiple testing. Alternatively, nonparametric tree-based predictive models, such as random forests (RFs) [2,3] have been suggested for the detection of unknown interactions, and were recommended as a pre-screening tool for large-scale association studies [4].

RFs consist of a collection of classification or regression trees grown from a random selection of variables on bootstrapped samples (bagging). The importance of a variable (average importance, AvImp) can be estimated by the increase in misclassification for the left-out (out-of-bag) samples when using a data vector containing the original variable values and a vector with randomly permuted values. It has been shown by simulation studies that RF importance measures are able to simultaneously detect single-locus heterogeneity models and multiplicative interaction models [4]. RF can also capture joint variable effects by a recently introduced joint-importance measure [5], which extends the concept of single importance by jointly permuting the values of variables of interest. However, the correspondence between LR interaction effects, defined as deviation from multiplicativity, and RF importance measures has not been explored yet.

Our study investigated the extent to which LR single-locus and pairwise interaction effects correspond to RF single- and joint-importance measures on a small data set of 20 markers (Genetic Analysis Workshop 15 (GAW15) rheumatoid arthritis (RA) candidate gene data set). Because RFs analysis cannot handle missing data [3], we also examined the sensitivity of both LR and RF analyses to different methods of data imputation. Finally, we investigated the efficacy of RFs as screening tool within a hypothetical two-stage design in which significant RF markers within our data set would be followed up by LR analysis.

## Methods

### Data selection

For our study we choose the GAW15 North American Rheumatoid Arthritis Consortium (NARAC) candidate gene data set (Problem 2) consisting of 20 biallelic markers (0 ≤ *r*^2 ^≤ 0.66) from at least 13 RA candidate genes residing within at least 10 different chromosomal locations; with on average one or two markers per gene. Because some cases within this data set originated from the same pedigree, independent individuals were selected at random to give 463 (out of the original 839) cases and 855 independent controls, all of Caucasian origin. Hardy-Weinberg equilibrium (HWE) was tested in the complete sample and in the controls. One rare single-nucleotide polymorphism (SNP), HugotSNP8ms2 (minor allele frequency <0.05), showed nominally significant deviations from HWE in the controls (*p *= 0.008) and in the full sample (*p *= 0.006) due to an overrepresentation of rare homozygotes, which might be the result of chance or genotyping error. This would have greatest effect on genotypic or haplotype analysis, neither of which was carried out within this study. All tests for HWE were non-significant after correction for multiple testing (20 markers).

### Imputation of missing values

Since RFs cannot handle missing values [3], data imputation was performed. Missing values in the original data set were either replaced by a RF strategy choosing the median of all marker alleles in the class of the missing value (median-replaced), or by imputation according to 'pseudo'-diplotype frequencies (imputed). Those frequencies were based on haplotype frequencies in the combined case-control sample, derived by an Expectation-maximization algorithm such as implemented in UNPHASED (v.2) [6]. Imputed marker genotypes were randomly selected from a multivariate uniform distribution. We included HugotSNP8ms2 in the imputation process since we observed low frequencies for haplotypes carrying the rare allele (<0.05), which are unlikely to affect the multivariate uniform distribution of diplotypes. Nine individuals with more than 50% missing genotypes were excluded as well as rs2240340, which had more than 65.4% dropouts. Missing values for the remaining markers ranged between less than 0.01% and 16.65%. In total, five imputed data sets were generated.

### Single-marker and pairwise interaction analysis using LR

Marker genotypes were recoded in terms of additive components [7] and single marker allelic effects and pairwise marker interactions investigated with LR, using the R program suite (v.2.4) [8]. LR models with large SEs (>3) were excluded. Adjustment for multiple testing of interaction terms was made by permutation tests (1000 permutations). For imputed data sets, LR estimates (based on Wald tests) were combined respectively as outlined by Rubin [9]; analyses of the full interaction model were combined using average differences in deviance between the model containing main and interaction effects, and the null model (χ^2^-test, 3 df).

### RF analysis of single- and joint-marker importance

**Figure 1:**
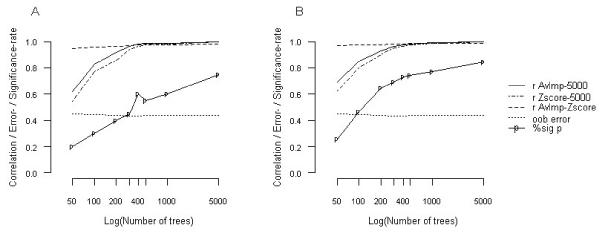
**Performance of RFs grown with different numbers of trees**. Performance of single (A) and joint (B) importance measures for RFs grown on 50, 100, 200, 300, 400, 500, 1000, and 5000 trees, respectively (median-replaced data). r AvImp-5000, Correlation of AvImps of each RF compared to RFs based on 5000 trees; r Zscore-5000, Correlation of *Z*-scores of each RF compared to RFs based on 5000 trees; r AvImp-Zscore, Correlation between AvImp and *Z*-score for each RF; %sig p, Percentage of significant AvImps. All RF features apart from the tree number were held constant.

The predictive importance of single markers [3] and marker pairs [5] was measured by *Z*-scores and significance levels. *Z*-scores can be inferred from AvImp measures through division by their associated standard error (SE), which is determined by the AvImp measure and the number of trees grown [3]. AvImp measures increase with the number of trees and their rank becomes stable, provided the number of trees is sufficiently large, such as observed by Lunetta et al. [4]. The associated *Z*-scores and their significance, however, also continue to increase with increasing number of trees, even when the RF reaches stability (see Figure 1). We investigated the correlation between AvImp measures for RFs with 5000 trees and RFs with 50, 100, 200, 300, 400, 500, and 1000 trees, respectively (median-replaced data); and in a similar way, the correlation between *Z*-scores (Spearman rank correlation). We observed high correlations (*r*_AvImp-5000 _≥ 0.98, *r*_Zscore-5000 _≥ 0.96) for RFs with ~500 trees and more, for both single and joint AvImp measures (see Figure 1), implying a) a stable rank among their most important markers and b) that significant markers identified by these RFs remain significant when analyzed using RF grown on 5000 trees. We therefore selected stable but small RFs (500 trees), which give similar AvImp ranks as RFs grown on 5000 trees.

Joint-importance analysis was implemented in the RF program (v.5.1) [3] based on the original Fortran code provided by A. Bureau (personal communication). Genotypes were coded as outlined for LR analysis. When trees were grown, the best split at each node was selected, which most efficiently reduced the out-of-bag error (balanced for cases and controls). Importance measures for imputed data sets were combined using AvImp estimates and pooled variances.

## Results

**Figure 2:**
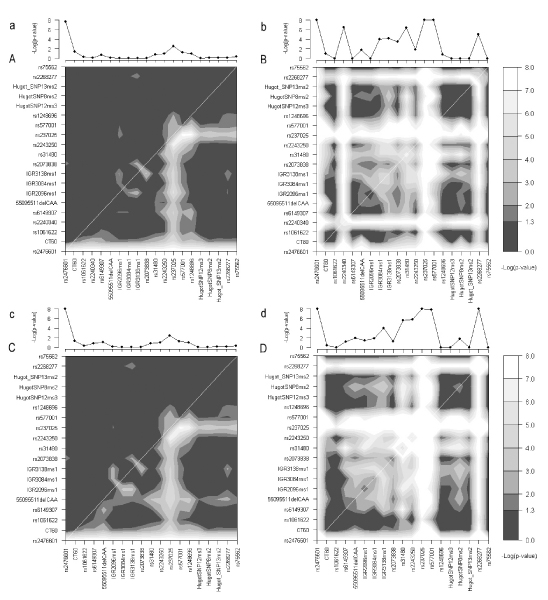
**Comparison between LR analysis and RF importance measures**. a/A, Single marker additive (a) and pairwise interaction effects (A) by LR using the median-replaced data. Upper left of A depicts the interaction-specific analysis, lower right the full LR interaction model. b/B, RF single (b) and joint (B) marker importance analysis based on 500 trees using the median-replaced data. Both triangles of B are identical. c/C, same as a/A using five imputed data sets (rs2240340 was excluded). d/D, same as b/B using five imputed data sets (rs2240340 was excluded). Markers with *p *≤ 10^-8 ^were truncated to *p *= 10^-8^; All presented *p*-values are uncorrected for multiple testing.

In the following section, the most significant nominal *p*-values for the median-replaced data set are reported in the text. In addition, nominal *p*-values for both median-replaced and imputed data are represented within Figure 2.

### Single-marker and pairwise interaction analysis using LR

Single-marker analysis showed highly significant disease association with rs2476601 (*p *= 2 × 10^-8^), and nominally significant effects for rs237025 (*p *= 0.003) and CT60 (*p *= 0.038) in both the median-replaced and the imputed data sets (see Figure 2a and 2c). The most significant pairwise interactions were found between the markers rs237025–rs577001 (*r*^2 ^= 0.62, *p *= 0.002), IGR3138ms1-rs2073838 (*r*^2 ^= 0.044, p = 0.008), IGR2096ms1–IGR3138ms1 (*r*^2 ^= 0.27, *p *= 0.008) and rs237025–rs1248696 (*r*^2 ^< 0.01, *p *= 0.009) using both median-replaced and imputed data (see Figure 2A and 2C). The LR models for the low-frequency markers HugotSNP12ms3-HugotSNP8ms2 and HugotSNP12ms3-Hugot_SNP13ms2 were excluded due to large SEs. In total, 12 pairs with nominally significant interactions (10 in imputed data) were identified, representing 6% of all marker combinations. This did not include the rare SNP HugotSNP8ms2, which showed deviations from HWE. Single marker and interaction analysis of the original data set with missing values showed similar results (data not shown). None of the interactions remained significant after correction for multiple testing using permutation tests (data not shown). Nominally significant full interaction models were observed for 47–53% of all marker combinations (median-replaced vs. imputed data) (see Figure 2A and 2C).

### RF analysis of single- and joint-marker importance

The average out-of-bag error for RFs was 44% (44–45% in imputed data) for both cases and controls. RF analysis identified 11 SNPs (12 in imputed data) with significant single importance (see Figure 2b and 2d), of which 8 markers (9 in imputed data) also showed nominally significant LR single locus effects or pairwise interactions: rs2476601 (p_RF _≤ 1 × 10^-16^) and rs237025 (p_RF _= 4.6 × 10^-10^) corresponded to LR single marker and interaction effects. IGR3084ms1, IGR3138ms1, rs2073838, rs2243250, rs577001, rs2268277, and in imputed data sets also IGR2096ms1 (5.6 × 10^-14 ^≤ p_RF _≤ 0.014), were involved in pairwise LR interactions (see Figure 2A and 2b; C and 2d). The remaining markers with significant single importance showed no correspondence to LR effects. Also, the allelic association with CT60 or interactions with rs1248696 were not reflected by the single-importance measure.

Large numbers of significant joint importance measures for SNP pairs were identified using both median-replaced (73%) and imputed data sets (70%) and represented predominantly markers with significant single-importance measures (see Figure 2B and 2C). The detected joint importance measures comprised all nominally significant LR interaction pairs (see Figure 2A and 2B; C and 2D) and 82 to 87% (median-replaced vs. imputed data) of the nominally significant full models.

## Discussion

Our study explored the comparability of parametric (LR) with nonparametric (RFs) analysis techniques when investigating the presence of single-locus and pairwise effects between 20 markers of the NARAC candidate gene data set. Both RF single- and joint-importance measures selected most of the markers for which nominally significant allelic or pairwise interaction effects by LR were detected. RF joint variable importance measures also captured most of the nominally significant full LR models, containing both main (i.e., multiplicative) and interaction effects (i.e., deviations from multiplicativity). This correspondence between LR and RFs was improved when analysis was performed with imputed instead of median-replaced data sets, which agrees with the results of Bureau et al. [5], and could probably be further increased if data imputation were based only on multiple markers from the same genomic region. However, because data imputation based on diplotype frequencies can be biased by deviations from HWE, the underlying marker genotype distribution needs to be carefully investigated.

The most consistent pairwise effect was obtained between the 6q25.1 markers rs237025 and rs577001, for which nominally significant interactions were found by LR as well as significant single and joint RF importance measures. rs237025 also showed nominally significant single-locus effects. Both rs237025 and rs577001 reside within the intronic region of *MAP3K7IP2*, but rs237025 also causes a non-synonymous change (M55V) within the reverse transcribed *SUMO4 *[10]. Although statistical interaction does not imply biological interaction, our results could implicate an epistatic effect between *SUMO4 *and *MAP3K7IP2*, which might increase susceptibility for RA. The significance of the statistical effect remains to be evaluated. Whereas LR permutation tests did not indicate the presence of any overall significant interaction, model-free RF analyses, which are robust to noisy variables and overfitting [2,3], provided evidence for highly significant single locus and joint effects.

The number of markers/marker pairs selected by RF measures was considerably larger than the number of pairs with significant LR interactions (6%) or significant LR full models (47–53%). Some of these might be false positives. However, it might also reflect the ability of RFs to detect a wide range of interaction models including multiplicative or heterogeneity effects [4], which might be accumulated in a candidate gene data set such as investigated in this study, whereas LR interaction analyses test the contribution of loci to disease risk specifically as departures from a multiplicative model.

Model-free analysis by RFs requires further investigation in order to identify high-risk allele or genotype combinations, and parametric follow-up analysis by LR represents one of several follow-up options. Choosing a hypothetical two-stage design, pairwise LR analysis of variables with significant RF single importance would have detected 90% of all nominally significant LR interactions in our small data set while analyzing only 39% of all possible marker combinations. LR analysis of all marker pairs with significant joint importance measures would have detected all nominally significant LR interaction effects while analyzing 70% of all possible combinations. This suggests that RF single-importance measures, which are able to detect a wide range of interaction effects [4] and are computationally very efficient [2], might be exploited as pre-screening tool for larger association studies, which aim for exhaustive two locus searches [1], although the minimum number of trees required for a RF to be a stable screening tool in those studies remains to be investigated. Joint RF importances showed even higher power to detect interactions effects, including combined LR main (multiplicative) and interaction effects, and might have increased power to detect disease-associated SNPs in discrete pre-specified subsets of data [5]. However, as observed by Bureau et al. [5] they require more computational time and might be less applicable as a screening tool.

In contrast to the approach suggested by Lunetta et al. [4], the efficacy of a *Z*-score based RF screening will be affected by selection of RFs with inflated numbers of trees, as these RFs show an increased number of significant signals (see Figure 1). If as suggested by Lunetta et al. [4], SNP ranking is used to select markers, the number of trees used to construct the RF is less important; though ranking of SNPs does not provide a measure of significance, which can be used for selection, whereas *Z*-scores do.

## Conclusion

Our study found a strong correspondence between RF importance measures and LR interaction effects, which was improved when data imputation was based on genetic data. The most consistent pairwise effect on RA was observed between two markers within *MAP3K7IP2/SUMO4 *on 6q25.1, although both LR and RFs assigned different significance levels. RF single-importance measures, which are able to detect a wide range of interaction effects and are computationally very efficient, might be also exploited as pre-screening tool for larger association studies, which aim for exhaustive two-locus searches. Joint-importance measures, although very powerful, might be less suitable for screening as they require more computational time. The approach of selecting markers based on their significance level using small but stable RFs (500 trees) was sufficient for the analysis of this small data set, but needs to explored for larger studies.

## Competing interests

The author(s) declare that they have no competing interests.
